# How the surgical treatment of lung cancer in the UK has evolved over the last two decades- An illustrative surgeon's experience

**DOI:** 10.1186/1749-8090-10-S1-A18

**Published:** 2015-12-16

**Authors:** Rocco Bilancia, Amit Paik, Annabel Sharkey, David Waller

**Affiliations:** 1 Thoracic Surgery, Glenfield Hospital, Leicester, LE3 9QP, UK

## Background/Introduction

The status of lung cancer surgery in UK has seen many changes over the last 20 years, with innovations in surgical technique and investigatory modalities together with significant organisational changes.

## Aims/Objectives

To assess how these changes have impacted on an individual surgical practice spanning this era.

## Method

We have retrospectively reviewed a single-surgeon practice from consultant appointment to present (1997-2015). We studied 1717 consecutive lung cancer operations: 962 lobectomy, 250 extended lobectomy, 57 pneumonectomy 296 sublobar, 43 open/close. Additionally, 710 surgical staging procedures were performed. We analysed trends with time in type of procedure; approach used (VATS/Open); open/close rates and in-hospital mortality.

## Results

1566 anatomic resections were performed (87 cases/year, 67-130). The following trends were observed:

1) Related to the disease itself

- A significant decrease in pneumonectomy rates (p < 0.001)

- An inversely proportional, increasing use of sleeve-resections (p = 0.088).

2) Related to surgical technique

- An increasing number of anatomical segmentectomies (p < 0.001)

- Stable rates of non-anatomical wedge resections (mean 6.3%, p = 0.908)

- An increasing proportion of VATS resection, both for lobectomies (p < 0.001) and segmentectomies (34.1 vs.14.6%, p < 0.001).

3) Related to healthcare system

- A significant decrease in use of surgical mediastinal staging, particularly after 2010 (p < 0.001)

- A significant decrease in-hospital mortality (mean 5.8%, p = 0.004)

- A significant reduction of open/close rates, particularly after 2004 (4.8 vs.0.65%, p < 0.001).

## Discussion/Conclusion

There has been significant evolution in lung cancer surgery over the last two decades, which is reflected in this individual surgeon's practice. Whilst increased surgical experience may partly explain the changes, most important factors include: a change in the proportion of central squamous and peripheral adenocarcinomas; earlier tumour detection, facilitating more VATS and lung-sparing surgery; improved perioperative care and use of lesser resections, reducing mortality; new techniques in staging (CT-PET,EBUS) reducing the need for surgical staging and the number of futile thoracotomies.

